# Contemporary Role of Open Left Thoracoabdominal Approach in Esophageal Malignancy Treatment

**DOI:** 10.7759/cureus.62922

**Published:** 2024-06-22

**Authors:** Dionysios Dellaportas, Ioannis Margaris, Panagiotis Latsonas, Anastasia Pikouli, Iakovos Vlachos, Dimitrios Papakonstantinou, Nikolaos Pararas, Constantinos Nastos, Despoina Myoteri, Emmanuel Pikoulis

**Affiliations:** 1 3rd Department of Surgery, Attikon University Hospital, National and Kapodistrian University of Athens, Athens, GRC; 2 4th Department of Surgery, Attikon University Hospital, National and Kapodistrian University of Athens, Athens, GRC; 3 Department of Pathology, Aretaieion University Hospital, National and Kapodistrian University of Athens, Athens, GRC

**Keywords:** malignant esophageal condition, esophageal surgery, esophagectomy, gastroesophageal cancer, esophageal carcinoma

## Abstract

Introduction: Despite the widespread use of minimally invasive techniques, open left thoracoabdominal esophagectomy (LTE) can offer excellent visualization and adaptability. The current study aimed to present and evaluate the outcomes related to an open LTE in esophageal malignancy treatment.

Methods: A retrospective cohort analysis of data collected from two institutions was performed, including patients with distal esophageal or junctional tumors who underwent open LTE between November 2018 and December 2023.

Results: Twenty-two patients were included (16 males; mean age 62.8 years). Postoperative complications occurred in eight patients (36%), with pulmonary complications being the most prevalent (seven patients; 32%). One patient experienced a clinical anastomotic leak. No reoperations or escalation to a higher level of care were required. In-hospital and 30-day mortality were zero. Tumor cells were found at the surgical margins in six patients (27%). The mean lymph node yield was 27. During the follow-up period, there were nine deaths and 11 cases of disease recurrence. Isolated locoregional recurrence was seen in five patients (23%). The one-year and two-year overall survival rates were 79% and 47%, respectively.

Conclusion: In selected cases, open LTE remains a valid and safe operation with acceptable morbidity and oncological efficacy.

## Introduction

The combination of neoadjuvant oncological therapy and surgery with radical lymphadenectomy is considered the cornerstone of treatment for resectable esophageal and gastroesophageal cancer [1]. Large studies have failed to provide definite evidence that one single surgical procedure is superior to another [2,3]. Hence, the optimal surgical strategy is a matter of debate, and decision-making is individualized. Specific tumor characteristics such as the exact location, longitudinal extension, lymph nodal status, and bulk of the primary neoplasm, as well as the patient’s anatomy and comorbidities, are taken into account whilst selecting the surgical approach.

Minimally invasive techniques are applied with rising incidence in esophageal surgery and have been associated with improved short-term and comparative long-term oncological results to the open techniques [4]. However, open approaches are still valid and are widely performed [5]. In certain clinical scenarios, a left thoracoabdominal esophagectomy (LTE) might be preferable for tumors of the distal esophagus or the gastroesophageal junction (GEJ) mainly because of its versatility, excellent exposure and safety [6,7]. This study aims to present and evaluate our experience with open LTE, along with the merits of this less-used surgical procedure for esophageal malignancy treatment.

## Materials and methods

Study design

A retrospective cohort study from two institutions performing esophageal surgery was conducted. Patients with distal esophageal or GEJ malignant tumors who underwent an open LTE between November 2018 and December 2023 were included. All operations were performed by the same surgical team. Patient and tumor characteristics, operative details, and postoperative outcomes were compiled. The study was conducted in accordance with the Declaration of Helsinki's ethical principles for medical research.

Outcomes measured and definitions used

The primary outcomes were postoperative morbidity and mortality and measures of the oncological efficacy of the operation, including margin status, lymph node yield, and follow-up oncological results. Complications were recorded using the definitions and recommendations provided by the Esophagectomy Complications Consensus Group (ECCG) and scored according to the Clavien-Dindo classification [8,9]. Circumferential resection margin (CRM) status was based on the definition used by the College of American Pathologists for tumor presence at the surgical resection margin [10]. Survival was calculated from the date of surgery to the date of death from any cause. Surviving patients were censored at the date of their last follow-up visit. Recurrence was clinically suspected but radiologically, endoscopically, or histologically verified. The follow-up schedule included outpatient visits every three to six months for the first two years postoperatively and yearly thereafter.

Data were also collected for patients’ baseline characteristics, operative time, length of hospital stay, and additional pathological variables. Surgical specimens were examined by dedicated upper gastrointestinal pathologists. The latest 8th edition manual of the American Joint Committee on Cancer (AJCC) was used for staging esophageal and esophagogastric junction tumors [10]. Siewert’s classification was applied for junctional tumors based on the evaluation of the surgical specimens [11]. To assess tumor response after neoadjuvant treatment, Mandard tumor regression scores were utilized [12].

Staging protocol

Patients were staged with the use of esophagogastroduodenoscopy (EGD), computed tomography (CT) of the chest, abdomen, and pelvis, and fluorodeoxyglucose positron emission tomography (FDG PET) CT. Selectively, endoscopic ultrasound (EUS) and staging laparoscopy were also employed. After completion of neoadjuvant treatment, patients were restaged before receiving surgical treatment with curative intent.

Systematic therapy

Most of the patients received perioperative chemotherapy with 5-Fluorouracil (5-FU), leucovorin, oxaliplatin, and docetaxel (FLOT regimen) [13]. Alternatively, preoperative chemoradiation was administered, based on the results of the Chemoradiotherapy for Oesophageal Cancer Followed by Surgery Study (CROSS) [14]. Adjuvant fluoropyrimidine-based chemoradiation therapy was selectively given to patients who had not received any kind of preoperative treatment or those who had residual disease after the operation. Since 2021, adjuvant nivolumab was considered for select patients, based on the results of the CheckMate 577 trial [15]. 

Surgical approach

All patients were intubated using a double-lumen endotracheal tube. Thoracic epidural analgesia was routinely used. The patient's position was a right lateral decubitus with a slight posterior tilt. The oblique incision was initiated as a laparotomy from the mid-epigastric area towards the costal arch. Following abdominal exploration, the incision was extended similar to a left posterolateral thoracotomy. The sixth intercostal space was used for entry into the thoracic cavity. The cartilage of the costal arch was sharply divided using rib cutters. The left hemidiaphragm was circumferentially incised. A subtotal esophagectomy with subtotal gastrectomy and en block lymphadenectomy was performed to include the tumor with at least 5 cm proximal and distal clearance. Abdominal lymphadenectomy included the paracardial, lesser curvature, left gastric artery, celiac trunk, common hepatic artery, and splenic artery nodes. During the thoracic phase, a middle and lower mediastinal clearance was performed, including the paraoesophageal, supradiaphragmatic, posterior mediastinal, subcarinal, and left main bronchus lymph nodes. For reconstruction, an end-to-side esophagogastric anastomosis at the level of the aortic arch was fashioned by inserting a 25-mm circular stapling device through a separate gastrotomy. A chest drain was inserted to drain both pleural spaces bibasally. A feeding jejunostomy tube was placed at the discretion of the surgeon. Layered closure was then performed with the use of heavily braided, non-absorbable sutures. Patients were extubated and transferred to a common surgical ward.

Statistical analysis

Continuous variables are presented as mean with standard deviation (±SD) or median with interquartile range (IQR), according to data distribution. Normality was assessed by using the one-sample Kolmogorov-Smirnov test. Categorical variables are expressed as counts and percentages. For statistical analysis, IBM Corp. Released 2021. IBM SPSS Statistics for Windows, Version 28.0. Armonk, NY: IBM Corp. was used.

## Results

During the study period, 22 patients, including 16 males and six females, underwent an open left thoracoabdominal esophagectomy for malignant tumors located at or around the GEJ. Baseline characteristics of the study population are presented in Table 1. The mean age of the patients at the time of the operation was 62.8 (± 8.8) years. Average Charlson Comorbidity Index (CCI) was 4.6 (± 1.6). Most of them (64%) had an American Society of Anesthesiologists (ASA) score of II. Twenty-one patients (95%) had a tumor invading beyond the muscularis propria of the esophagus, as evidenced by the preoperative staging modalities used. Nodal involvement was also present in 95% of the included patients. Fifteen patients (68%) received neoadjuvant treatment, either chemotherapy or chemoradiotherapy.

**Table 1:** Baseline characteristics of the study population Data are presented as counts (%) or mean (± SD)

Baseline characteristics			
Age (years); mean (± SD)		62.8 (±8.8)	
Sex	Female	6 (27%)	
	Male	16 (73%)	
Charlson Comorbidity Index; mean (± SD)		4.6 (± 1.6)	
ASA score	I	3 (14%)	
	II	14 (64%)	
	III	5 (23%)	
	IV - V	0	
Body Mass Index; mean (± SD)		26.26 (± 6.14)	
Clinical tumor stage	T1	0	
	T2	1 (5%)	
	T3	18 (82%)	
	T4	3 (14%)	
Clinical nodal stage	N0	1 (5%)	
	N1	14 (64%)	
	N2	5 (23%)	
	N3	2 (9%)	
Neoadjuvant treatment	None	7 (32%)	
	Chemotherapy	12 (55%)	
	Chemoradiotherapy	3 (14%)	

All patients underwent an en-block esophageal-gastrectomy with two-field lymphadenectomy. Total gastrectomy was not required in any case, and the anastomosis was always placed at the subaortic plane. The mean operative time was 212 (± 20.4) minutes. A feeding jejunostomy tube was inserted in 16 patients (73%). The mean length of hospital stay (LOS) was 11 (± 3.1) days. For the majority of patients, there was no deviation from the normal postoperative course. However, eight patients (36%) suffered from postoperative complications (Table 2). Pulmonary complications developed in seven cases (32%). Severe complications (Clavien-Dindo ≥3) occurred in three patients (14%). There was a single anastomotic leak type II, which was diagnosed 30 days postoperatively. The patient was readmitted due to intermittent fevers, paroxysmal cough, and blood-tinged sputum. He was found to have a small broncho-esophageal fistula on contrast swallow, which was successfully treated with an endoscopic stent insertion and appropriate antibiotic coverage. None of the patients required a return to theatre for reexploration or admission to the intensive care unit. Thirty-day and in-hospital mortality were zero (Table 2).

**Table 2:** Postoperative morbidity and mortality ^† ^Pneumonia defined as a new lung infiltrate on chest X-ray or computed tomography, along with infectious signs, such as fever, leukocytosis, purulent sputum, and hypoxemia. ^‡^The patient with anastomotic leak was also found to have a lower respiratory tract infection, due to a small bronchoesophageal fistula.

Postoperative morbidity and mortality			
Complications	Total	8 (36%)	
Pulmonary complications		7 (32%)	
	Pneumonia^†^	5 (23%)	
	Pneumothorax requiring drainage	1 (5%)	
	Chest effusion requiring drainage	1 (5%)	
Cardiovascular	Atrial dysrhythmia requiring treatment	1 (5%)	
Anastomotic leak	Type II	1 (5%)^‡^	
Clavien-Dindo classification	0 & I	14 (64%)	
	II	5 (23%)	
	IIIa	2 (9%)	
	IIIb	1 (5%)	
	IV & V	0	
In-hospital/30-day mortality		0	

The results of the final histopathological parameters are presented in Table 3. On final pathology, 86% of the patients had an adenocarcinoma of the GEJ. Siewert II adenocarcinoma, located 1 cm above to 2 cm below the GEJ, represented the most common type. All patients underwent a potentially curative resection. Nonetheless, six of them (27%) were subsequently found to have tumor cells at the resection margin. A positive circumferential resection margin (CRM) was the most common scenario. Residual microscopic disease was found in the longitudinal resection margins in three patients (14%). In two of them, a positive longitudinal margin coexisted with a positive CRM. Tumor length on histological examination was available for 20 patients, and the mean value was 5.37 (± 2.72) cm. The mean distance of the proximal end of the tumor to the proximal resection margin, available for 17 patients, was 3.46 (± 1.62) cm. After neoadjuvant treatment, there was no complete pathologic response (pCR) on the evaluation of the resected specimens. Most of the patients had advanced tumor or nodal stage on histopathological assessment; 77% of the patients had ≥pT3 tumors, while 55% of the patients had pN2/N3 disease. The mean number of lymph nodes retrieved was 27 (± 9.90). The median (IQR) number of positive lymph nodes was 3 (0.75-7.00).

**Table 3:** Final histopathological parameters Data are presented as counts (%), median (IQR) or mean (± SD)

Final histopathological parameters			
Histology	Adenocarcinoma	19 (86%)	
	Adenosquamous carcinoma	1 (5%)	
	Squamous cell carcinoma	2 (9%)	
Siewert type	I	4 (18%)	
	II	15 (68%)	
	III	3 (14%)	
Lymphovascular invasion	Yes	18 (82%)	
	No	4 (18%)	
Mandard tumor regression grade	1-2	1 (5%)	
	3-4	7 (32%)	
	5	2 (9%)	
	Not available	5 (23%)	
	Not applicable	7 (32%)	
Clear resection margins	Yes (R0)	16 (73%)	
	No (R1)	6 (27%)	
	Proximal R1	2 (9%)	
	Distal R1	1 (5%)	
	Circumferential R1	5 (23%)	
Tumor length (cm); mean (± SD)		5.37 (± 2.72)	
Distance from proximal margin (cm); mean (± SD)		3.46 (± 1.62)	
Pathological tumor stage	T0	0	
	T1	1 (5%)	
	T2	4 (18%)	
	T3	11 (50%)	
	T4	6 (27%)	
Pathological nodal stage	N0	5 (23%)	
	N1	5 (23%)	
	N2	6 (27%)	
	N3	6 (27%)	
Lymph nodes retrieved	0-10	0	
	11-20	8 (36%)	
	21-30	4 (18%)	
	˃30	10 (45%)	
Lymph node yield; mean (± SD)		27 (± 9.90)	
Number of positive lymph nodes; median (IQR)		3 (0.75-7.00)	

The average follow-up time was 18.3 (± 12.9) months. The one-year and two-year overall survival rates were 79% and 47%, respectively. During the follow-up period, there were nine deaths, while 11 patients experienced a disease recurrence. Isolated locoregional recurrence occurred in five patients (23%). Distant recurrence, either isolated or in conjunction with local recurrence, developed in six patients; one patient presented with peritoneal dissemination, two patients developed liver metastases, and three patients had a distant lymphatic spread.

## Discussion

The primary goal of our study was to examine the feasibility of an open left thoracoabdominal approach in terms of postoperative morbidity, mortality, and oncological efficacy. Our results confirm that the major morbidity from an esophagectomy stems from pulmonary complications. Nonetheless, postoperative mortality was zero. On histological examination, six patients (27%) had tumor presence at the surgical margins, while a mean number of 27 lymph nodes were retrieved. The cohort’s survival rate at one year was 79%.

The optimal surgical strategy for esophageal and GEJ malignancies remains largely controversial. However, transthoracic approaches seem to offer the best chances for a potentially curative resection [16]. Even though minimally invasive esophagectomy (MIE) techniques offer distinct advantages, open procedures remain valid and are widely performed around the world [5]. An open LTE may prove particularly useful for patients with bulky junctional tumors, obese patients, or when previous laparotomies and chest infections are anticipated to hinder dissection, owing to the presence of dense adhesions [7,17]. In such cases, a single incision gains access to both the abdominal and the thoracic cavities and provides a great view of the diaphragmatic crura and the lower esophagus, which anatomically deviates towards the left side. Furthermore, the semi-lateral position efficiently exposes the left gastric and short gastric vessels and facilitates gastric mobilization [6].

One of the possible concerns regarding the LTE is the presumed increased risk for pulmonary complications related to the thoracotomy wound itself and the division of the diaphragm. In our study, 23% of the patients developed postoperative pneumonia, which is concordant with previous reports for open transthoracic approaches [18,19]. However, all of them were successfully treated with antibiotics, and none of the patients developed respiratory failure, requiring a step up to a higher level of care. Klevebro et al. recently investigated the short-term outcomes related to either an open LTE or an MIE in 915 patients with esophageal cancer [20]. There was no difference in overall complications, pulmonary complications, severe complications, anastomotic leak, or in-hospital mortality between the two groups. We believe that, for several reasons, a left thoracoabdominal approach may lead to a less painful and morbid insult than generally assumed. Contrary to a standard right posterolateral thoracotomy, the left thoracoabdominal incision cuts through one or two dermatomes [17]. Additionally, instead of forcefully retracting the ribs, the costal cartilage is sharply divided. The diaphragm is circumferentially divided, avoiding a potential injury to the branches of the phrenic nerve. Finally, careful reapproximation of the costal arch and the routine use of epidural analgesia may also aid in reducing postoperative pain and its grave implications on the incidence of pulmonary complications.

Only a single patient in our study suffered from a clinical anastomotic leak. All esophageal-gastrectomies were fashioned at the level of the aortic arch, which is situated only a few centimeters distal to the level commonly used in right-sided intrathoracic anastomoses. Furthermore, the gastric conduit was easily pulled up and placed deep into the posterior mediastinum by following the straighter left-sided pathway. Hence, in all cases, a well-vascularized and tension-free gastric conduit was anastomosed, and this may account for the low rate of anastomotic complications observed.

The microscopic presence of tumor cells at the resection margins has been associated with tumor recurrence and reduced overall survival [21]. Positive circumferential resection margins (CRM) have been reported in as many as 10-20% of the patients in recent esophagectomy series [22,23]. Achieving a negative CRM may be particularly troublesome due to the esophageal proximity to important anatomical structures and the lack of any clear fascial margin. According to our results, 23% of the patients had a positive CRM, which was higher than expected. However, most of them had advanced tumor stages. Furthermore, 32% of our patients did not receive any form of preoperative systematic treatment, which could have increased the rate of R0 resections [13,14]. Even though our study lacks long-term follow-up data, the mid-term results provided can be indicative of the oncological efficacy of the operation. They are also clinically relevant, bearing in mind that most of the disease recurrences occur early after surgery, usually within a two-year timespan, which is a major limitation of long-term survival [24]. Our calculated one-year survival rate of 79% corresponds well to the reported results from large population-based studies and recent esophagectomy series from high-volume centers [7,25,26].

Another possible concern regarding the left thoracoabdominal operation is the ability to achieve a proximal clear margin since the presence of the aortic arch may limit the extent of resection. Indeed, two of our patients were found to have a positive proximal resection margin. It has been suggested that extending the dissection superiorly and performing a supra-aortic or cervical anastomosis is feasible and can diminish this problem with limited added morbidity [17,27]. In a recent comparative study, including 1228 patients with esophageal or EGJ tumors, there was no difference in the rate of positive resection margins between a left thoracoabdominal and a right thoracoabdominal (Ivor-Lewis) approach [7]. The anastomosis in the LTE group of patients was placed below or above the aortic arch or in the left cervical region. There was also no difference in long-term overall survival, suggesting that the two surgical approaches are related to equivalent oncological outcomes. In our opinion, the left thoracoabdominal is an extremely versatile option since dissection may extend proximally or distally, as required. As already mentioned, a higher-placed thoracic anastomosis will likely eliminate the risk for residual microscopic disease at the proximal resection margin. Additionally, when the distal margin seems to be threatened, surgical strategy can be adapted accordingly to include a total gastrectomy and a modification to a jejunal reconstruction [17].

A mean number of 27 lymph nodes were retrieved in our study, which correlates well with similar reports regarding the LTE approach [7,20]. Current guidelines still recommend a minimum of 15 nodes removed for adequate staging, even though the number may be different from that necessary for optimal survival [28]. Extend lymphadenectomy for GEJ tumors remains a matter of debate. Recent evidence suggests that type I junctional tumors carry a significant risk for metastasis to the upper mediastinal field, whereas for true cardia (type II) or subcardial (type III) tumors the risk is lower than 10%, and so the extent of lymph nodal dissection should be tailored based on the esophageal invasion length [29,30]. In the latter case, we consider that a left thoracoabdominal operation may well achieve negative resection margins and perform an adequate clearance of the lower and middle mediastinum. On the other hand, lower esophageal tumors or type II tumors with extensive esophageal involvement (>4cm) might be better served with a right transthoracic approach, which can readily provide access to the superior mediastinum, achieve an R0 resection, and clear the upper thoracic paraoesophageal and paratracheal stations.

Limitations

The current study suffers from significant limitations, and results need to be interpreted with caution. This was a small retrospective analysis of patients stemming from two institutions and operated by a single surgical team. Hence, confounding and selective reporting may have biased our results and hampered generalizability. Furthermore, other patient-important outcomes, including long-term complications or quality of life, were not assessed. Yet, we believe our results can shed light on this less frequently applied surgical approach and may be useful to guide decision-making.

## Conclusions

In selected cases, open LTE remains a valid and safe operation with acceptable morbidity and oncological efficacy. Our results indicate that in certain clinical scenarios, a left thoraco-laparotomy can effectively serve as a radical esophageal cancer operation. At the same time, it can also provide an excellent operative field and flexibility in dissection and reconstruction.
